# Neochamaejasmin A Induces Mitochondrial-Mediated Apoptosis in Human Hepatoma Cells via ROS-Dependent Activation of the ERK1/2/JNK Signaling Pathway

**DOI:** 10.1155/2020/3237150

**Published:** 2020-01-20

**Authors:** Yangfang Ding, Qi Xie, Wenjing Liu, Zhaohai Pan, Xinmei Fan, Xiaoyu Chen, Mingkai Li, Wei Zhao, Defang Li, Qiusheng Zheng

**Affiliations:** ^1^School of Integrated Traditional Chinese and Western Medicine, Binzhou Medical University, Yantai, 264003 Shandong, China; ^2^Jiangsu College of Nursing, Huaian, 223005 Jiangsu, China; ^3^Institute of Pharmaceutical Engineering, Jiangsu Food & Pharmaceutical Science College, Huaian, 223003 Jiangsu, China; ^4^Key Laboratory of Xinjiang Endemic Phytomedicine Resources of Ministry of Education, School of Pharmacy, Shihezi University, Shihezi, 832002 Xinjiang, China

## Abstract

The botanical constituents of *Stellera chamaejasme* Linn. exhibit various pharmacological and medicinal activities. Neochamaejasmin A (NCA), one main active constituent of *S. chamaejasme*, inhibits cell proliferation and induces cell apoptosis in several types of tumor cells. However, the antitumor effect of NCA on hepatocellular carcinoma cells is still unclear. In this study, NCA (36.9, 73.7, and 147.5 *μ*M) significantly inhibited hepatoblastoma-derived HepG2 cell proliferation in a concentration-dependent manner. Hoechst 33258 staining and flow cytometry showed that apoptotic morphological changes were observed and the apoptotic rate was significantly increased in NCA-treated HepG2 cells, respectively. Additionally, the levels of Bax, cleaved caspase-3, and cytoplasmic cytochrome *c* were increased, while the level of Bcl-2 was decreased in NCA-treated HepG2 cells when compared with the control group. Moreover, we found that the reactive oxygen species (ROS) level was significantly higher and the mitochondrial membrane potential was remarkably lower in NCA-treated HepG2 cells than in the control group. Further studies demonstrated that the levels of p-JNK and p-ERK1/2 were significantly upregulated in NCA-treated HepG2 cells, and pretreatment with JNK and ERK1/2 inhibitors, SP600125 and PD0325901, respectively, suppressed NCA-induced cell apoptosis of HepG2 cells. In addition, NCA also significantly inhibited human hepatoma BEL-7402 cell proliferation and induced cell apoptosis through the ROS-mediated mitochondrial apoptotic pathway. These results implied that NCA induced mitochondrial-mediated cell apoptosis via ROS-dependent activation of the ERK1/2/JNK signaling pathway in HepG2 cells.

## 1. Introduction

Hepatocellular carcinoma (HCC) is the most commonly occurring solid cancer. According to global cancer statistics, there were 841,080 new cases of liver cancer and 781,631 deaths in 2018 [1]. HCC is characterized by rapid and abnormal cell differentiation, rapid infiltration and growth, and early transition. Additionally, the development of highly malignant tumors and the accompanying poor prognosis are considered to be features of HCC [2, 3]. At present, surgery is considered to be the staple cure for HCC [4]. However, during surgery, an amount of liver tissue is removed, resulting in the inability of residual liver tissue to survive after surgery, and surgical treatment can only be a palliative treatment for metastatic liver cancer. Therefore, it has become the focus of research to try to find a new drug for hepatocellular carcinoma.

*Stellera chamaejasme* Linn. is a traditional Chinese herbal medicine in China. Moreover, a few studies have proved that the botanical constituents of *Stellera chamaejasme* inhibit the growth of several types of cancer cells, including human breast cancer MDA-MB-231 cells, human osteosarcoma MG63 cells, human lung carcinoma NCI-H157 cells, and human leukemia K562 cells [5–9]. Further studies showed that two active constituents (chamaejasmenin B and neochamaejasmin C) exert proliferation inhibitory effects on several human tumor cell lines, e.g., liver carcinoma HepG2 and SMMC-7721 cells, non-small cell lung cancer A549 cells, osteosarcoma cell MG63 and KHOS cells, and colon cancer cell HCT-116 cells [10].

**Figure 1:**
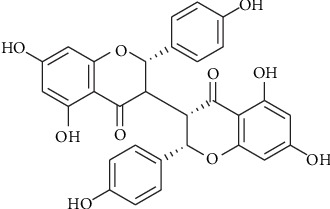
Structure of NCA.

A recent study reported that neochamaejasmin A (NCA, Figure 1), another main constituent in the dried root of *Stellera chamaejasme*, also exerts antitumor effects on tumor cells [11]. Liu et al. demonstrated that NCA induces cell cycle arrest at the G_1_ phase by activating p21 and subsequently promotes cell apoptosis via the Fas/caspase-8/caspase-3 pathway [11]. However, the anticancer effect of NCA on hepatocellular carcinoma cells has not yet been explored. In this study, we found that NCA possessed a robust activity against hepatocellular carcinoma HepG2 cells.

## 2. Materials and Methods

### 2.1. Materials

NCA was obtained from Wuhan ChemFaces (purity ≥ 98%). Dimethyl sulfoxide (DMSO) and 3-(4,5-dimethylthiazol-2-yl)-2,5-diphenyltetrazolium bromide (MTT) were provided from Sigma. Minimum essential medium (MEM) was obtained from Gibco. The reactive oxygen species (ROS) assay kit, mitochondrial membrane potential assay kit, and Annexin V-FITC/propidium iodide (PI) detection kit were provided by Nanjing KeyGen Biotech. Fetal bovine serum (FBS) was provided by TransGen Biotech Corporation. The Hoechst 33258 stain, penicillin-streptomycin, and cell lysates were obtained from Solarbio. Superoxide dismutase (SOD, Cat No. BC0175) and catalase (CAT, Cat No. BC0205) detection kits were provided by Beijing Solarbio Science & Technology Co., Ltd. The JNK inhibitor SP600125 (Cat No. S1460) and ERK1/2 inhibitor PD0325901 (Cat No. S1036) were purchased from Selleckchem Corporation. Anti-JNK (Cat No. #9252), anti-phospho-JNK (Thr183/Tyr185, Cat No. #4668), ERK1/2 (Cat No. #4695), and anti-phospho-ERK1/2 (Thr202/Tyr204, Cat No. # 9101) were obtained from Cell Signaling Technology. Anti-caspase-3 (Cat No. ab4051) and anti-cleaved caspase-3 (Cat No. ab2302) were obtained from Abcam. Anti-*β*-actin and secondary antibodies were provided by Beijing ZSGB Biotechnology. The remainder of the antibodies were obtained from Cell Signaling Technology.

### 2.2. Cell Culture

Human hepatoblastoma-derived HepG2 cells and human hepatocellular carcinoma BEL-7402 cells were provided by the Chinese Academy of Sciences Cell Bank. The HepG2 cells were incubated in MEM medium with 10% FBS and 1% penicillin-streptomycin and cultured in a 37°C incubator with 5% CO_2_. The BEL-7402 cells were incubated in RPMI-1640 medium with 10% FBS and 1% penicillin-streptomycin and cultured in a 37°C incubator with 5% CO_2_.

### 2.3. Cell Viability Assay

The viabilities of HepG2/BEL-7402 cells were determined with the MTT assay [12–14]. The HepG2/BEL-7402 cell suspensions were inoculated into a 96-well plate with 100 *μ*L per well. After 24 h incubation, the cells were treated with different concentrations of NCA. Based on this situation that the final concentration of DMSO used to dissolve NCA is 0.05%, the complete medium containing with 0.05% DMSO was used and chosen as the control group. After cells were exposed to NCA (for HepG2 cells, 18.4, 36.9, 73.7, and 147.5 *μ*M; for BEL-7402 cells, 50, 100, 150, 200, 250, and 300 *μ*M) for 24, 48, and 72 h in 96-well plates with 1 × 10^5^ cells/mL, the original medium was replaced with fresh medium and 10 *μ*L prepared MTT (5 mg/mL) solution was added to the HepG2/BEL-7402 cells for another 4 h. Then, the medium was removed, and 150 *μ*L DMSO was added per well to dissolve insoluble formazan crystals. A microplate reader measured the absorbance at 570 nm (Thermo Fisher Scientific, Inc., Waltham, MA, USA).

### 2.4. Morphological Assay

Morphological changes in HepG2/BEL-7402 cells were observed using Hoechst 33258 staining. The cells were treated with NCA (for HepG2 cells, 36.9, 73.7, and 147.5 *μ*M; for BEL-7402 cells, 100, 150, and 200 *μ*M) on 6-well chamber slides for 48 h. Cells were treated with a fixative solution (the ratio of methanol to glacial acetic acid is 3 : 1) for 15 minutes protected from light. After a certain time, HepG2 cells were washed and soaked in Hoechst 33258 (10 mg/L) to stain for 10 minutes at 37°C protected from light, as previously described [15]. The cell morphology was observed with fluorescence microscopy (Carl Zeiss, Germany).

### 2.5. Reactive Oxygen Analysis

The DCFH-DA probe was applied to detect ROS levels in HepG2 cells [16]. After treatment with NCA (for HepG2 cells, 36.9, 73.7, and 147.5 *μ*M; for BEL-7402 cells, 100, 150, and 200 *μ*M) for 48 h, the HepG2 cells were stained for 30 min at 37°C using 30 *μ*M DCFH-DA protected from light, and then the HepG2 cells were washed and resuspended in phosphate-buffered saline (PBS). The final treated cells were detected by flow cytometry (BD Biosciences).

### 2.6. Detection of Intracellular Oxidoreductase SOD and CAT

The activities of SOD and CAT in HepG2 cells were examined using SOD and CAT kits. Briefly, the cells were exposed to NCA (for HepG2 cells, 36.9, 73.7, and 147.5 *μ*M; for BEL-7402 cells, 100, 150, and 200 *μ*M) for 48 h, and then SOD and CAT were tested according to SOD and CAT kits, respectively. For SOD detection, the cell lysis solutions (18 *μ*L) were added in a 96-well microtiter plate and subsequently 45 *μ*L test solution 1, 2 *μ*L test solution 2, 35 *μ*L test solution 3, 90 *μ*L test solution 4, and 10 *μ*L test solution 5 per well were added. After mixing well, the plate was incubated for 30 min at 37°C. Finally, the absorbance values were detected at 560 nm with a microplate reader (Thermo Fisher Scientific, Inc., Waltham, MA, USA). For CAT detection, the test working solution was preheated at 37°C for 10 min. Then, 10 *μ*L cell lysis solution and 190 *μ*L test working solution were added into a 96-well microtiter plate. Finally, the initial absorbance value (A1) and the absorbance value after 1 min of reaction (A2) were detected at 240 nm with a microplate reader. The activities of SOD and CAT were calculated according to the instruction of the SOD and CAT kits.

### 2.7. Cell Apoptosis Analysis

The apoptosis rate of HepG2/BEL-7402 cells was analyzed by flow cytometry [17]. The HepG2/BEL-7402 cell suspensions were inoculated into a 96-well plate with 100 *μ*L per well. After incubation for 24 h, the cells were treated with different concentrations of NCA (for HepG2 cells, 36.9, 73.7, and 147.5 *μ*M; for BEL-7402 cells, 100, 150, and 200 *μ*M) with/without the JNK inhibitor SP600125 (5 *μ*M) or the ERK1/2 inhibitor PD0325901 (1 *μ*M). Then, the cells were digested and washed with PBS twice, and 5 *μ*L Annexin FITC and 5 *μ*L PI were added to the HepG2 cells for 10 minutes. After that, the apoptosis rate was determined by flow cytometer (FACSCalibur; BD Biosciences).

### 2.8. Mitochondrial Membrane Potential Analysis

The HepG2/BEL-7402 cells were treated with NCA (for HepG2 cells, 36.9, 73.7, and 147.5 *μ*M; for BEL-7402 cells, 100, 150, and 200 *μ*M) for 48 h, and then cells were collected and exposed to JC-1 (5,5′,6,6′-tetrachloro-1,1′,3,3′-tetraethylbenzimidazolyl carbocyanine iodide) solution for 20 minutes in the dark at a temperature of 37°C. Cells were detected by flow cytometry (FACSCalibur; BD Biosciences) and microplate reader (Thermo Fisher Scientific, Inc, Waltham, MA, USA) at excitation/emission wavelengths of 485/580 nm for red and 485/530 nm for green [18].

### 2.9. Western Blot Analysis

HepG2 cells were exposed to NCA (73.7 *μ*M) with/without the JNK inhibitor SP600125 (5 *μ*M) or the ERK1/2 inhibitor PD0325901 (1 *μ*M) for 48 h; cell lysates were added to HepG2 cells on ice for 30 minutes, and then the total protein from the cell supernatants was harvested. The protein underwent separation by 15% sodium dodecyl sulfate-polyacrylamide gel electrophoresis (SDS-PAGE), and then the proteins were electrotransferred to polyvinylidene difluoride (PVDF) membranes. Next, the membranes with protein were blocked with 5% nonfat milk or bovine serum albumin (BSA) in Tris-buffered saline (TBS) containing 0.1% Tween-20 (TBST) solution for 2 h, and the membranes were subsequently incubated with specific primary antibodies at 4°C overnight. Next, the membranes were incubated with the corresponding secondary antibodies for 1 h, and the bands on the membranes were visualized by the use of the enhanced chemiluminescence (ECL) kit (Thermo Fisher Scientific) and the EC3 imaging system (Spring Scientific, USA). The bands of the Western blot were then analyzed using ImageJ software (NIH, Bethesda, Maryland, USA) [19, 20]. The results were standardized with *β*-actin.

### 2.10. Statistical Analysis

The data are expressed as the mean ± standard deviation from at least three experiments. The data were analyzed by one-way analysis of variance (ANOVA) followed by Fisher's multiple comparison test using SPSS 21.0 software (Chicago, IL, USA). *P* < 0.05 was used to evaluate if the difference is statistically significant.

## 3. Results

### 3.1. NCA Inhibits HepG2 Cell Proliferation and Induces Cell Morphology Changes

**Figure 2:**
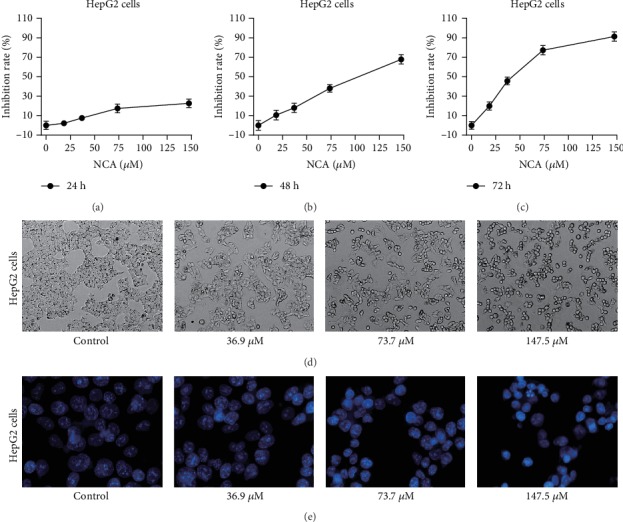
NCA inhibited HepG2 cell proliferation and induced changes in cell morphology. (a–c) HepG2 cells were exposed to NCA for 24, 48, and 72 h, respectively, and the cell viability was determined by MTT assay. (d) After NCA treatment for 48 h, the morphological changes of HepG2 cells were observed under a phase contrast microscope. (e) Hoechst 33258 staining was used to examine the morphological change of HepG2 cells after exposure to NCA for 48 h. ^∗^*P* < 0.05 and ^∗∗^*P* < 0.01, compared with the control group.

To observe the antitumor effect of NCA on HepG2 cells, the MTT assay was employed to test the sensitivity of HepG2 cells. We found that NCA significantly inhibited HepG2 cell proliferation in a concentration-dependent manner (Figures 2(a)–2(c)). When the concentration of NCA reached 147.5 *μ*M, the inhibition rate reached 22.6%, 67.8%, and 91.4% after 24, 48, and 72 h of treatment, respectively. After treatment with NCA for 48 h, the cells began to shrink when compared with the control group (Figure 2(d)). Next, Hoechst 33258 staining was used to evaluate the morphological changes in NCA-treated HepG2 cells. Chromatin condensation and apoptotic bodies were also observed in HepG2 cells (Figure 2(e)).

### 3.2. NCA Induces HepG2 Cell Apoptosis and Regulates the Levels of Apoptosis-Related Proteins

**Figure 3:**
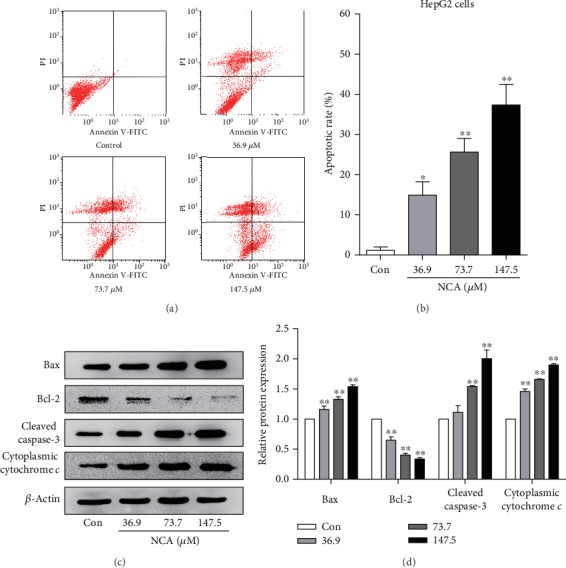
NCA induced HepG2 cell apoptosis and regulated the apoptosis-associated protein levels. (a) The apoptotic rate of NCA-treated HepG2 cells was determined by flow cytometry. (b) Statistical analysis of the apoptotic rate of NCA-treated HepG2 cells. (c, d) HepG2 cells were treated with NCA for 48 h, and the protein levels of Bax, cleaved caspase-3, and cytoplasmic cytochrome *c* were analyzed by Western blot. ^∗^*P* < 0.05 and ^∗∗^*P* < 0.01, compared with the control group.

In order to further confirm the effect of NCA on cell proliferation, Annexin V-fluorescein isothiocyanate (FITC)/propidium iodide (PI) staining was performed to explore whether NCA could induce apoptosis. After treatment with different concentrations of NCA (36.9, 73.7, and 147.5 *μ*M) for 48 h, the apoptosis rate of HepG2 cells was significantly increased when compared with the control group (Figures 3(a) and 3(b)). Then, we examined the levels of apoptosis-associated molecules in HepG2 cells using Western blotting. The data revealed that the protein levels of Bax, cleaved caspase-3, and cytoplasmic cytochrome *c* were significantly increased, while the level of Bcl-2 was significantly decreased in NCA-treated HepG2 cells when compared to those in the control group (Figures 3(c) and 3(d)).

### 3.3. NCA Induces a Mitochondrial-Dependent Apoptotic Pathway in HepG2 Cells

**Figure 4:**
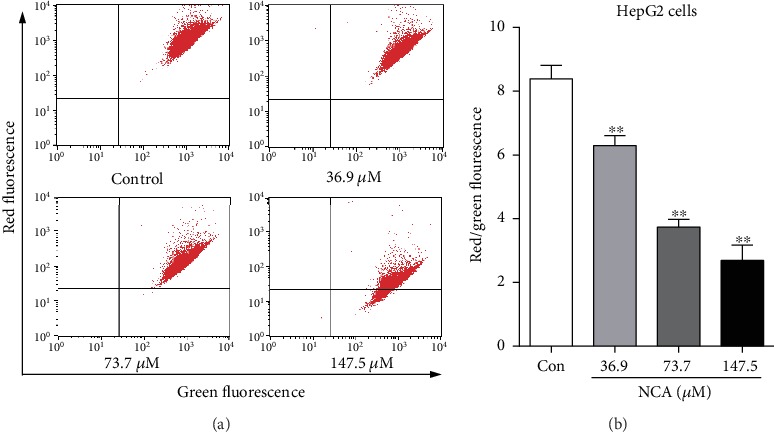
NCA induced changes in the mitochondrial membrane potential in HepG2 cells. (a) HepG2 cells were treated with NCA for 48 h, and the mitochondrial membrane potential changes were subsequently evaluated by JC-1 staining. (b) Statistical analysis of the ratio of red to green fluorescence in NCA-treated HepG2 cells. ^∗^*P* < 0.05 and ^∗∗^*P* < 0.01, compared with the control group.

At present, the mitochondrial pathway exerts a vital role in cell apoptosis [21–23]. To explore the key role of mitochondria in apoptosis, JC-1 dye was used to determine the change in the mitochondrial membrane potential in NCA-treated HepG2 cells. The results showed that the ratio of red to green fluorescence was significantly decreased in NCA-treated cells when compared with the control group (Figures 4(a) and 4(b)). It is implied that NCA triggered disorder in the mitochondrial membrane potential and subsequently induced the mitochondrial-dependent apoptotic pathway.

### 3.4. NCA Induces the Production of Reactive Oxygen Species (ROS) and Regulates the Activities of Antioxidant Enzymes

**Figure 5:**
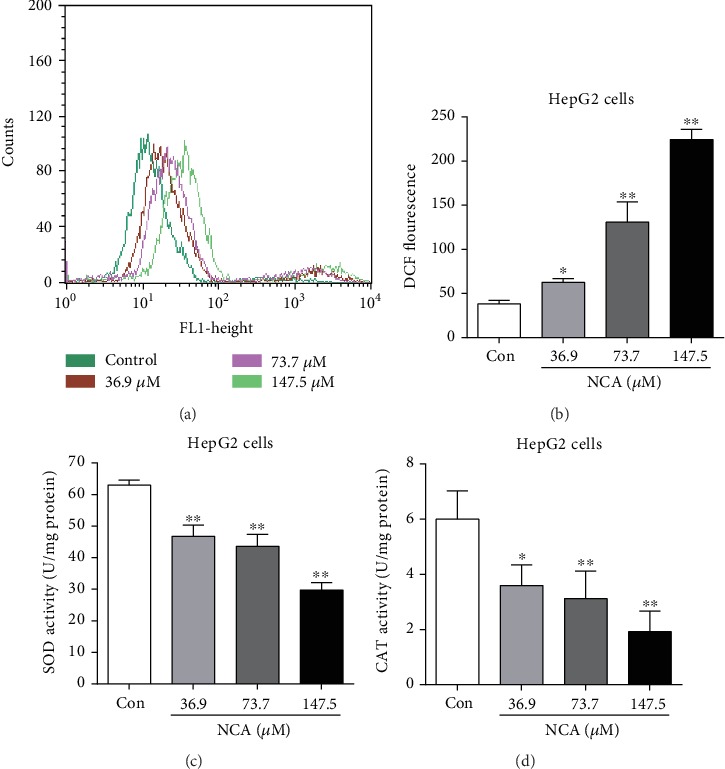
NCA caused ROS to be produced and downregulated the activities of antioxidant enzymes in HepG2 cells. (a) ROS production in NCA-treated HepG2 cells was determined using a DCFH-DA fluorescence probe. (b) Statistical analysis of the green fluorescence intensity in NCA-treated HepG2 cells. (c) The activity of SOD in NCA-treated HepG2 cells was detected with the SOD kit. (d) The activity of CAT in NCA-treated HepG2 cells was detected with the CAT kit. ^∗^*P* < 0.05 and ^∗∗^*P* < 0.01, compared with the control group.

Evidence indicated that the disorder of the mitochondrial membrane potential is inextricably linked to the level of ROS and the production of ROS affects the function of the mitochondrial membrane [24, 25]. Therefore, a 2′,7′-dichlorodihydrofluorescein diacetate (DCFH-DA) probe was employed to measure the level of ROS in HepG2 cells after treatment with NCA for 48 h. We found that the green fluorescence intensity of DCF was significantly increased after 48 h of treatment with NCA, implying that NCA induced ROS production in HepG2 cells (Figures 5(a) and 5(b)). Next, the activities of superoxide dismutase (SOD) and catalase (CAT) were measured for the purpose of observing changes in antioxidant enzymes when HepG2 cells were exposed to NCA for 48 h. When compared with the control group, the activities of SOD and CAT were significantly decreased, as expected (Figures 5(c) and 5(d)).

### 3.5. Effect of NCA on the ERK1/2/JNK Signaling Pathway in HepG2 Cells

**Figure 6:**
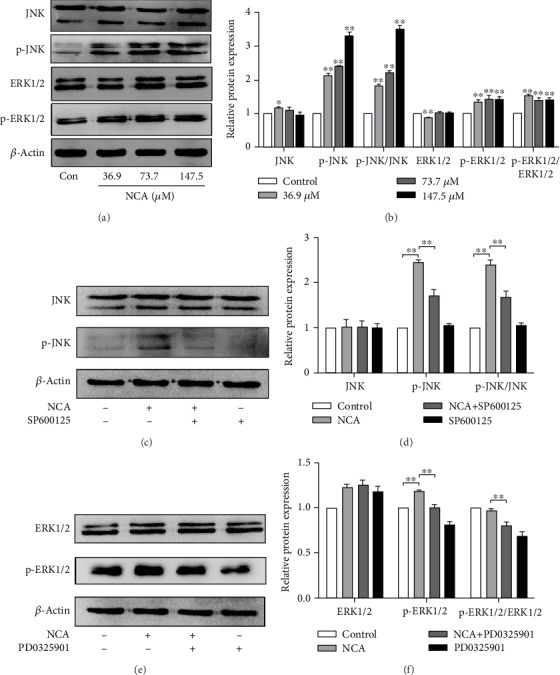
Effect of NCA on JNK/ERK signaling molecules in HepG2 cells. (a, b) HepG2 cells were treated with NCA for 48 h, and the levels of JNK, p-JNK, ERK1/2, and p-ERK1/2 were examined by Western blot. (c, d) HepG2 cells were pretreated with 10 *μ*M SP600125 for 1 h and subsequently treated with 73.7 *μ*M NCA for 48 h, and the levels of JNK and p-JNK were then examined by Western blot. (e, f) HepG2 cells were pretreated with 10 *μ*M PD0325901 for 1 h and subsequently treated with 73.7 *μ*M NCA for 48 h, and then the levels of ERK1/2 and p-ERK1/2 were examined by Western blot. ^∗^*P* < 0.05 and ^∗∗^*P* < 0.01, compared with the control group.

Some studies have shown that apoptosis is always linked to activation of the *ERK1/2/JNK* signaling pathway [26–28]. Western blot analysis demonstrated that the levels of p-JNK (Thr183/Tyr185) and p-ERK1/2 (Thr202/Tyr204) were increased in HepG2 cells after treatment with NCA for 48 h (Figures 6(a) and 6(b)). Furthermore, JNK and ERK1/2 inhibitors, SP600125 and PD0325901, respectively, were employed to explore the effect of NCA on the *ERK1/2/JNK* signaling pathway in HepG2 cells. The increased levels of p-JNK and p-ERK1/2 in NCA-treated HepG2 cells were significantly attenuated by pretreatment with SP600125 and PD0325901, respectively (Figures 6(c)–6(f)).

### 3.6. The ERK1/2/JNK Signaling Pathway Is Involved in NCA-Induced HepG2 Cell Apoptosis

**Figure 7:**
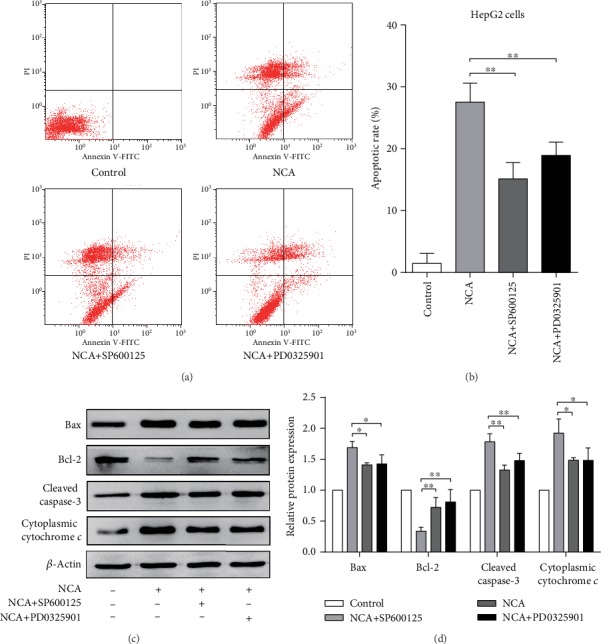
JNK and ERK1/2 are involved in HepG2 cell apoptosis induced by NCA. HepG2 cells were pretreated with 10 *μ*M SP600125 or PD0325901 for 1 h and then treated with 73.7 *μ*M NCA for 48 h. (a, b) The apoptotic rate was examined flow cytometry. (c, d) The protein levels of Bax, cleaved caspase-3, and cytoplasmic cytochrome *c* were analyzed by Western blot. ^∗^*P* < 0.05 and ^∗∗^*P* < 0.01, compared with the control group.

We next explored whether the *ERK1/2/JNK* signaling pathway is involved in HepG2 cell apoptosis induced by NCA. We found that the increased apoptotic rate in NCA-treated HepG2 cells was significantly attenuated by pretreatment with SP600125 or PD0325901 (Figures 7(a) and 7(b)). Additionally, the upregulated levels of Bax, cleaved caspase-3, and cytoplasmic cytochrome *c*, as well as the downregulated level of Bcl-2, were suppressed by pretreatment with SP600125 or PD0325901 (Figures 7(c) and 7(d)). These data revealed that the ERK1/2/JNK signaling molecules participated in NCA-induced HepG2 cell apoptosis.

### 3.7. NCA Induces Cell Apoptosis and Increases ROS Level in BEL-7402 Cells

**Figure 8:**
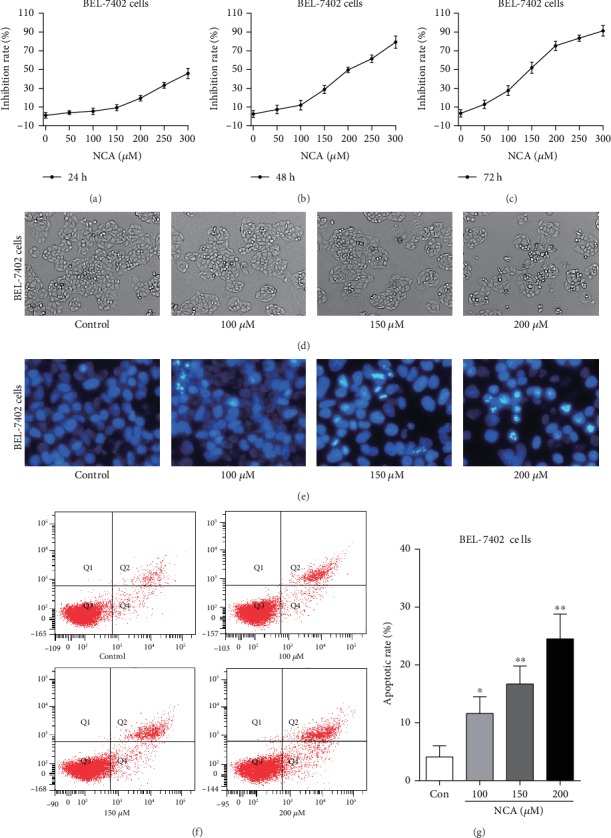
NCA inhibited cell proliferation and induced cell apoptosis in BEL-7402 cells. (a–c) BEL-7402 cells were exposed to NCA for 24, 48, and 72 h, respectively, and the cell viability was determined by MTT assay. (d) The morphological changes of NCA-treated BEL-7402 cells were observed under a phase contrast microscope. (e) Hoechst 33258 staining was used to examine the morphological change of BEL-7402 cells after exposure to NCA for 48 h. (f) The apoptotic rates of NCA-treated BEL-7402 cells were determined by flow cytometry. (g) Statistical analysis of the apoptotic rate of NCA-treated BEL-7402 cells. ^∗^*P* < 0.05 and ^∗∗^*P* < 0.01, compared with the control group.

**Figure 9:**
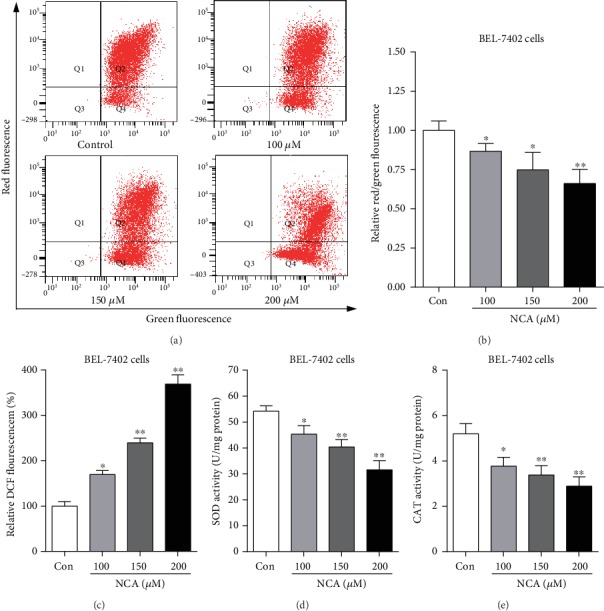
NCA induced changes in the mitochondrial membrane potential and ROS levels in BEL-7402 cells. (a) BEL-7402 cells were treated with NCA for 48 h, and the mitochondrial membrane potential changes were subsequently evaluated by JC-1 staining. (b) Statistical analysis of the ratio of red to green fluorescence in NCA-treated BEL-7402 cells. (c) Statistical analysis of the green fluorescence intensity in NCA-treated BEL-7402 cells. (d) The activities of SOD in NCA-treated BEL-7402 cells were detected with the SOD kit. (e) The activities of CAT in NCA-treated BEL-7402 cells were detected with the CAT kit. ^∗^*P* < 0.05 and ^∗∗^*P* < 0.01, compared with the control group.

Consistent with the data from HepG2 cells, we found that NCA significantly inhibited BEL-7402 cell proliferation after treatment with NCA for 24, 48, and 72 h (Figures 8(a)–8(c)). Meanwhile, the NCA-treated BEL-7402 cells began to shrink, and the chromatin condensation and apoptotic bodies were observed in BEL-7402 cells (Figures 8(d) and 8(e)). Flow cytometry analysis showed that the apoptosis rate of BEL-7402 cells was significantly increased when compared with the control group (Figures 8(f) and 8(g)). Furthermore, we found that the ratio of red to green fluorescence was significantly decreased in NCA-treated BEL-7402 cells when compared with the control group (Figures 9(a) and 9(b)). In addition, the green fluorescence intensity of DCF was significantly increased (Figure 9(c)), and the activities of SOD and CAT were significantly decreased in NCA-treated BEL-7402 cells (Figures 9(d) and 9(e)). These data implied that NCA also induced BEL-7402 cell apoptosis through the ROS-mediated mitochondrial-dependent apoptotic pathway.

## 4. Discussion

Hepatocellular carcinoma (HCC) is one of the malignant tumors that can endanger health and result in considerable fatality. Traditional chemotherapy is one of the methods to assist liver cancer treatment [29]. However, traditional chemotherapy has some worrying issues, such as high toxicity and minimal effect, in prolonging survival. Therefore, it is of great significance to find effective candidate compounds for curing liver cancer. Recently, numerous scientists have focused on compounds extracted from natural products, and it was found that NCA significantly inhibited several different types of tumor cells [11]. This prompted us to explore whether NCA could effectively suppress the cell proliferation and growth of hepatocellular carcinoma. In our research, NCA inhibited the proliferation of HepG2 and BEL-7402 cells in a concentration-dependent manner. In order to explore the mechanism that NCA uses to inhibit HepG2 and BEL-7402 cell proliferation, we observed the morphological changes in HepG2 and BEL-7402 cells after treatment with NCA and we noted that the NCA-treated cells began to shrink, with chromatin condensation and the formation of apoptotic bodies. In addition, the rate of apoptosis of NCA-treated HepG2 and BEL-7402 cells was significantly increased when compared with the control group. All of the data indicated that NCA inhibited HepG2 and BEL-7402 cell proliferation via inducing cellular apoptosis.

Previous studies revealed that excessive ROS triggered mitochondrial dysfunction, activated the MAPK pathway, and caused cellular apoptosis [30–32]. When tumor cells are stimulated by stress signals from outside or inside, it affects the Bcl-2 family of proteins, including an increase in protein Bax expression and a decrease in protein Bcl-2 expression. Then, due to mitochondrial membrane potential disorder, the mitochondrial membrane pores open and a large amount of cytochrome *c* is released to the cytoplasm, forming a complex with Apaf-1, which activates caspase-9, leading to active downstream caspase factor, which causes apoptosis [33–35]. Our results showed that NCA caused an increase in ROS production as well as a decrease in the activities of SOD and CAT in HepG2 and BEL-7402 cells. NCA also triggered mitochondrial membrane potential disorder, which caused a large amount of cytochrome *c* to be released to the cytoplasm, as well as upregulating the levels of Bax and cleaved caspase-3 and downregulating the level of Bcl-2 in HepG2 cells. Taken together, these results implied that NCA could induce HepG2 and BEL-7402 cell apoptosis via the ROS-mediated mitochondrial apoptotic pathway.

The ERK and JNK signaling pathways have been found in mammalian cells, and they are two parallel MAPK signaling pathways that are important in cell growth, differentiation, apoptosis, and other stress and inflammatory response effects [36, 37]. Evidence has shown that excessive ROS regulates mitochondrial pathway-induced cell death by activating the ERK1/2/JNK pathway and simultaneously activating molecules such as Bax, thereby leading to mitochondrial dysfunction and cell death [38]. In this process, activated JNK can activate nuclear transcription factors, such as c-fos, ATP-2, p53, c-Myc, and nontranscription factors, such as the Bcl-2 superfamily in apoptosis [39]. In addition, the activation of JNK can simultaneously change the mitochondrial membrane potential and the release of cytochrome *c*, which then causes a downstream cascade to induce apoptosis [40]. ERK1 and ERK2 are the two most important members of the ERK pathway [41].

In this study, the protein levels of p-JNK (Thr183/Tyr185) and p-ERK1/2 (Thr202/Tyr204) were remarkably increased in NCA-treated cells. To observe whether JNK and ERK1/2 play vital roles in apoptosis when HepG2 cells were exposed to NCA, JNK and ERK1/2 inhibitors SP600125 and PD0325901 were used. The results showed that SP600125 and PD06325901 reversed the upregulation of Bax and cleaved caspase-3, the downregulation of Bcl-2, the release of cytochrome *c*, and HepG2 cell apoptosis induced by NCA. Collectively, these results demonstrated that NCA could induce HepG2 cell apoptosis via ROS-dependent activation of the ERK1/2/JNK signaling pathway.

However, several limitations in this study should be noted. Firstly, the potential therapeutical target (protein) of NCA is still unclear. Second, the antitumor effects of NCA were examined in HepG2 and BEL-7402 cells, but the cytotoxicity of NCA in normal cells, e.g., L02 cells, was not determined. In addition, the effect of NCA in a hepatoma-xenografted mouse model has not been evaluated.

## 5. Conclusions

The data demonstrated that NCA significantly inhibited hepatocellular carcinoma cell proliferation and induced cell apoptosis via the mitochondrial apoptosis pathway, which was regulated by ROS-dependent activation of the ERK1/2/JNK signaling pathway.

## Data Availability

The data used to support the findings of this study are included within the article.

## References

[1] Bray F., Ferlay J., Soerjomataram I., Siegel R. L., Torre L. A., Jemal A. (2018). Global cancer statistics 2018: GLOBOCAN estimates of incidence and mortality worldwide for 36 cancers in 185 countries. *CA: A Cancer Journal for Clinicians*.

[2] Llovet J. M., Bruix J. (2008). Molecular targeted therapies in hepatocellular carcinoma. *Hepatology*.

[3] Stotz M., Gerger A., Haybaeck J., Kiesslich T., Bullock M. D., Pichler M. (2015). Molecular targeted therapies in hepatocellular carcinoma: past, present and future. *Anticancer Research*.

[4] Bruix J., Gores G. J., Mazzaferro V. (2014). Hepatocellular carcinoma: clinical frontiers and perspectives. *Gut*.

[5] Yang D., Wang P., Ren X. (2015). Apoptosis induced by chamaejasmine in human osteosarcoma cells through p53 pathway. *Tumour Biology*.

[6] Zhang T., Yu H., Dong G., Cai L., Bai Y. (2013). Chamaejasmine arrests cell cycle, induces apoptosis and inhibits nuclear NF-*κ*B translocation in the human breast cancer cell line MDA-MB-231. *Molecules*.

[7] Wang Y., Zhao Y., Liu Y., Tian L., Jin D. (2011). Chamaejasmine inactivates Akt to trigger apoptosis in human HEp-2 larynx carcinoma cells. *Molecules*.

[8] Liu X., Li Y., Yang Q. (2012). In vitro inhibitory and pro-apoptotic effect of Stellera chamaejasme L extract on human lung cancer cell line NCI-H157. *Journal of Traditional Chinese Medicine*.

[9] Zhang S. D., Shan L., Li W., Li H. L., Zhang W. D. (2015). Isochamaejasmin induces apoptosis in leukemia cells through inhibiting Bcl-2 family proteins. *Chinese Journal of Natural Medicines*.

[10] Zhang C., Zhou S. S., Feng L. Y. (2013). In vitro anti-cancer activity of chamaejasmenin B and neochamaejasmin C isolated from the root of *Stellera chamaejasme* L. *Acta Pharmacologica Sinica*.

[11] Liu W. K., Cheung F. W., Liu B. P., Li C., Ye W., Che C. T. (2008). Involvement of p21 and FasL in induction of cell cycle arrest and apoptosis by neochamaejasmin A in human prostate LNCaP cancer cells. *Journal of Natural Products*.

[12] Ma Y. T., Yang Y., Cai P. (2018). A series of enthalpically optimized docetaxel analogues exhibiting enhanced antitumor activity and water solubility. *Journal of Natural Products*.

[13] Liang Y., Tian B., Zhang J. (2017). Tumor-targeted polymeric nanostructured lipid carriers with precise ratiometric control over dual-drug loading for combination therapy in non-small-cell lung cancer. *International Journal of Nanomedicine*.

[14] Chi Y., Yin X., Sun K. (2017). Redox-sensitive and hyaluronic acid functionalized liposomes for cytoplasmic drug delivery to osteosarcoma in animal models. *Journal of Controlled Release*.

[15] Ren B., Ye L., Gong J. (2019). Alteronol enhances the anti-tumor activity and reduces the toxicity of high-dose adriamycin in breast cancer. *Frontiers in Pharmacology*.

[16] Chen X. Y., Ren H. H., Wang D. (2019). Isoliquiritigenin induces mitochondrial dysfunction and apoptosis by inhibiting mitoNEET in a reactive oxygen species-dependent manner in A375 human melanoma cells. *Oxidative Medicine and Cellular Longevity*.

[17] Pan Z., Qu C., Chen Y. (2019). Bufotalin induces cell cycle arrest and cell apoptosis in human malignant melanoma A375 cells. *Oncology Reports*.

[18] Liu W., Liu X., Pan Z. (2019). Ailanthone induces cell cycle arrest and apoptosis in melanoma B16 and A375 cells. *Biomolecules*.

[19] Yang Y., Guan D., Lei L. (2018). H6, a novel hederagenin derivative, reverses multidrug resistance in vitro and in vivo. *Toxicology and Applied Pharmacology*.

[20] Lv G., Sun D., Zhang J. (2017). Lx2-32c, a novel semi-synthetic taxane, exerts antitumor activity against prostate cancer cells in vitro and in vivo. *Acta Pharmaceutica Sinica B*.

[21] Márquez-Jurado S., Díaz-Colunga J., das Neves R. P. (2018). Mitochondrial levels determine variability in cell death by modulating apoptotic gene expression. *Nat Commun*.

[22] Singh P. K., Roukounakis A., Frank D. O. (2017). Dynein light chain 1 induces assembly of large Bim complexes on mitochondria that stabilize Mcl-1 and regulate apoptosis. *Genes & Development*.

[23] van Loo G., Saelens X., van Gurp M., MacFarlane M., Martin S. J., Vandenabeele P. (2002). The role of mitochondrial factors in apoptosis: a Russian roulette with more than one bullet. *Cell Death &Differentiation*.

[24] Vandenabeele P., Galluzzi L., Vanden Berghe T., Kroemer G. (2010). Molecular mechanisms of necroptosis: an ordered cellular explosion. *Nature Reviews Molecular Cell Biology*.

[25] Liu M. P., Liao M., Dai C. (2016). *Sanguisorba officinalis* L synergistically enhanced 5-fluorouracil cytotoxicity in colorectal cancer cells by promoting a reactive oxygen species-mediated, mitochondria-caspase-dependent apoptotic pathway. *Scientific Reports*.

[26] Li X., Han Y., Guan Y., Zhang L., Bai C., Li Y. (2012). Aluminum induces osteoblast apoptosis through the oxidative stress-mediated JNK signaling pathway. *Biological Trace Element Research*.

[27] Zhong F., Tong Z. T., Fan L. L. (2016). Guggulsterone-induced apoptosis in cholangiocarcinoma cells through ROS/JNK signaling pathway. *American Journal of Cancer Research*.

[28] Choi B. K., Choi C. H., Oh H. L., Kim Y. K. (2004). Role of ERK activation in cisplatin-induced apoptosis in A172 human glioma cells. *Neurotoxicology*.

[29] Schwartz J. D., Beutler A. S. (2004). Therapy for unresectable hepatocellular carcinoma: review of the randomized clinical trials—II: systemic and local non-embolization-based therapies in unresectable and advanced hepatocellular carcinoma. *Anti-Cancer Drugs*.

[30] Lin X., Wu S., Wang Q. (2016). Saikosaponin-D reduces H_2_O_2_-induced PC12 cell apoptosis by removing ROS and blocking MAPK-dependent oxidative damage. *Cellular and Molecular Neurobiology*.

[31] Gomez-Lazaro M., Galindo M. F., Melero-Fernandez de Mera R. M. (2007). Reactive oxygen species and p38 mitogen-activated protein kinase activate Bax to induce mitochondrial cytochrome c release and apoptosis in response to malonate. *Molecular Pharmacology*.

[32] Li S., Dong P., Wang J. (2010). Icariin, a natural flavonol glycoside, induces apoptosis in human hepatoma SMMC-7721 cells via a ROS/JNK-dependent mitochondrial pathway. *Cancer Letters*.

[33] Martinou J. C., Green D. R. (2001). Breaking the mitochondrial barrier. *Nature Reviews Molecular Cell Biology*.

[34] Zamzami N., Kroemer G. (2001). The mitochondrion in apoptosis: how Pandora's box opens. *Nature Reviews Molecular Cell Biology*.

[35] Parone P. A., James D., Martinou J. C. (2002). Mitochondria: regulating the inevitable. *Biochimie*.

[36] Sun Y., Liu W. Z., Liu T., Feng X., Yang N., Zhou H. F. (2015). Signaling pathway of MAPK/ERK in cell proliferation, differentiation, migration, senescence and apoptosis. *Journal of Receptor and Signal Transduction Research*.

[37] Shao Y., Wang C., Hong Z., Chen Y. (2016). Inhibition of p38 mitogen-activated protein kinase signaling reduces multidrug transporter activity and anti-epileptic drug resistance in refractory epileptic rats. *Journal of Neurochemistry*.

[38] Kim B. C., Kim H. G., Lee S. A. (2005). Genipin-induced apoptosis in hepatoma cells is mediated by reactive oxygen species/c-Jun NH_2_-terminal kinase-dependent activation of mitochondrial pathway. *Biochemical Pharmacology*.

[39] Liu J., Lin A. (2005). Role of JNK activation in apoptosis: a double-edged sword. *Cell Research*.

[40] Chauhan D., Li G., Hideshima T. (2003). JNK-dependent release of mitochondrial protein, Smac, during apoptosis in multiple myeloma (MM) cells. *Journal of Biological Chemistry*.

[41] Zhang X., Ma L., Qi J., Shan H., Yu W., Gu Y. (2015). MAPK/ERK signaling pathway-induced hyper-O-GlcNAcylation enhances cancer malignancy. *Molecular and Cellular Biochemistry*.

